# Frailty and nutritional inadequacy in older Korean adults: A gender-stratified analysis using National Survey Data

**DOI:** 10.1371/journal.pone.0333620

**Published:** 2025-10-27

**Authors:** Subeen Kim, Haerang Lee, Minji Kang

**Affiliations:** 1 Department of Food and Nutrition, Duksung Women’s University, Seoul, Republic of Korea; 2 BNK Management Research Institute, BNK Financial Group, Seoul, Republic of Korea; National Trauma Research Institute, AUSTRALIA

## Abstract

While frailty has traditionally been conceptualized through physical decline, it is increasingly recognized as a complex concept encompassing emotional, psychological, and social factors. This study employed a multidimensional framework to investigate the association between nutritional status and frailty levels across genders. In addition, it aims to provide foundational insights for developing targeted dietary and preventive health policies that support interventions tailored to the characteristics of specific older adult populations. This is a cross-sectional study of the 2009–2020 Korean National Health and Nutrition Examination Survey in 14,242 participants aged 65 and older. The frailty index was constructed using 41 items. Dietary data were obtained through a 24-hour dietary recall, and adequacy of nutrient intake was evaluated based on the Dietary Reference Intakes for Koreans 2020. Multivariable logistic regression analysis was conducted to examine the association between nutritional status and frailty levels. Among participants, 31.6% were categorized as non-frail, 47.8% as pre-frail, and 20.6% as frail. Women exhibited lower total energy intake and higher frailty prevalence than men. Gender-stratified analyses revealed distinct nutritional patterns: frail men showed a significant decreasing trend in riboflavin intake (P-trend = 0.0012), while frail women had increased carbohydrate (P-trend = 0.005) and decreased fat (P-trend = 0.0032) and riboflavin (P-trend = 0.0062) intake. Frailty significantly associated with iron inadequacy in men (OR=1.49, 95% CI:1.15–1.94; P-trend = 0.0018) and riboflavin inadequacy in women (OR=1.45, 95% CI:1.20–1.74; P-trend<0.0001). Frailty in older adults is associated with multidimensional vulnerabilities-including demographic, behavioral, relational, and nutritional factors-with notable gender differences in nutrient intake patterns. These findings underscore the need for gender-specific and integrated nutritional interventions to effectively prevent frailty and improve quality of life in the elderly population.

## Introduction

Later life is a distinct developmental stage in which individuals engage in reflection on their overall lives, seek existential meaning, integrate life experiences, and undergo emotional development as they approach the final phase of life [1]. With advancements in medical technology extending life expectancy and prolonging the later stages of life, the health and quality of life of older adults have emerged as central issues in global aging policy. During this period, older adults not only face physical decline but also experience cognitive deterioration and social role losses triggered by life events such as retirement [2]. Therefore, understanding aging requires a multidimensional approach that includes not only physical and medical perspectives but also economic, leisure, relational, residential, and occupational domains [3,4]. Successful aging is characterized by the absence of disease, maintenance of high cognitive and physical functioning, and active engagement in life across physical, psychological, and social domains [5].

Frailty, as distinct from the normal aging process, refers to a vulnerable state caused by a decline in physiological reserves, which diminishes the body’s ability to maintain homeostasis and respond to stressors [6,7]. Although frailty is sometimes broadly referred to as a vulnerable condition, it is increasingly recognized as a geriatric syndrome due to its clinical profile, which includes unintentional weight loss, muscle weakness, fatigue, reduced physical activity, and slowness of gait [8]. Frailty is not only associated with limitations in daily functioning but also contributes significantly to reduced quality of life in old age [9,10]. Its prevalence increases with advancing age and has been identified as a major predictor of disease incidence, mortality, falls, and hospitalization [11,12].

Frailty is influenced by various sociodemographic and lifestyle-related factors, including income, education, physical activity, social connectedness, and nutritional status. Prior studies have either focused on the validation of frailty measurement instruments [13–15] or explored associations between frailty and variables such as household composition, nutritional adequacy, comorbidities, and exercise patterns [16,17]. Among these, nutrition plays a particularly significant role in the onset and progression of frailty. Older adults with diminished physical function may face difficulties with food intake or medication adherence, and dietary interventions have been identified as a low-cost and effective method of preventing or delaying frailty [18–20]. Nutritional improvements are also expected to produce broader socioeconomic benefits, including reductions in national healthcare expenditures and improvements in population-level well-being [21,22].

In Korea, research on frailty has predominantly focused on the evaluation of Korean frailty measurement indices [23], the association between frailty and chronic diseases [24–26], and the relationship between frailty and nutritional status [27,28]. These studies suggest the need for further segmentation of frailty groups [25,28] and for a multidimensional frailty measurement index that also considers the emotional aspects of older adults [26]. Despite growing interest in frailty, there remains a lack of multidimensional research that simultaneously addresses the emotional, psychological, and nutritional aspects of frailty among older adults in Korea, as highlighted by previous studies. Therefore, in order to design accurate interventions tailored to the population group, it would be more meaningful and practical to assess the nutritional status of the elderly by segmenting them according to frailty level.

Meanwhile, although frailty-related research has accumulated both within Korea and internationally, most studies have concentrated on model development and validation [16,17]. Limited attention has been given to gender- or household-related differences in frailty, or to how nutritional status varies by frailty severity. Understanding the underlying causes of frailty is vital for prevention. Moreover, investigating frailty levels in conjunction with sociodemographic and nutritional variables is essential for tailoring public health strategies and market policies to distinct target populations, enhancing both policy effectiveness and implementation efficiency.

Traditionally, frailty has been measured through either the frailty phenotype (FP), which emphasizes physical performance indicators such as weight loss, grip strength, exhaustion, and low physical activity [9], or the frailty index (FI), which assesses the proportion of accumulated health deficits in an individual [29]. However, given the vulnerability of frail individuals to external stress due to reduced physiological reserves, it is increasingly acknowledged that emotional and psychological factors must also be considered. Recent research has thus expanded frailty measurement to encompass emotional and social domains, incorporating quality-of-life indicators such as the EuroQol-5 Dimension and mental health measures [13,30,31].

As the global aging population continues to rise, the urgency of frailty-related research grows. According to the United Nations [32,33], individuals aged 65 and older accounted for 19.8% of the global population in 2023, a figure projected to increase to 38.9% by 2100. Notably, the proportion of adults aged 80 and above is expected to surge significantly. In South Korea, the proportion of older adults reached 18.4% in 2023 and is expected to exceed 20.6% by 2025, signaling its transition into a super-aged society [34]. Given the rapid pace of demographic aging in South Korea, identifying the current state and drivers of frailty is of national and global relevance, providing a reference point for other aging societies.

This study aims to investigate the frailty levels and associated nutritional status of older adults in South Korea. Specifically, it extends the traditional focus on physical frailty to include emotional and psychological dimensions, allowing for a more comprehensive and multidimensional analysis of the condition. In response to previous calls for more nuanced approaches, this study further identifies distinct subgroups within the frail population based on varying degrees and types of frailty. It also examines how nutritional status varies by gender and by frailty level. The findings are expected to provide foundational evidence for developing targeted dietary strategies and preventative interventions, ultimately supporting the design of more personalized programs and policies for diverse subpopulations of older adults in both clinical and community settings.

## Materials and methods

### Study design and participants

This study analyzed data from the Korean National Health and Nutrition Examination Survey (KNHANES) collected between 2009 and 2020. The KNHANES is a nationally representative, cross-sectional survey conducted by the Korea Disease Control and Prevention Agency (KDCA), using a complex, multi-stage probability sampling method. The study protocol was approved by the Institutional Review Board (IRB) of KDCA (approval numbers: 2009-01CON-03-2C, 2010-02CON-21-C, 2011-02CON-06-C, 2012-01EXP-01-2C, 2013-07CON-03-4C, 2013-12EXP-03-5C, 2018-01-03-P-A, 2018-01-03-C-A, 2018-01-03-2C-A). However, in accordance with the bioethics and safety act, the study was exempted from IRB review in the years 2015, 2016, and 2017. Detailed information on the survey procedures is available on the KNHANES website https://knhanes.kdca.go.kr/knhanes/main.do [35].

**Fig 1:**
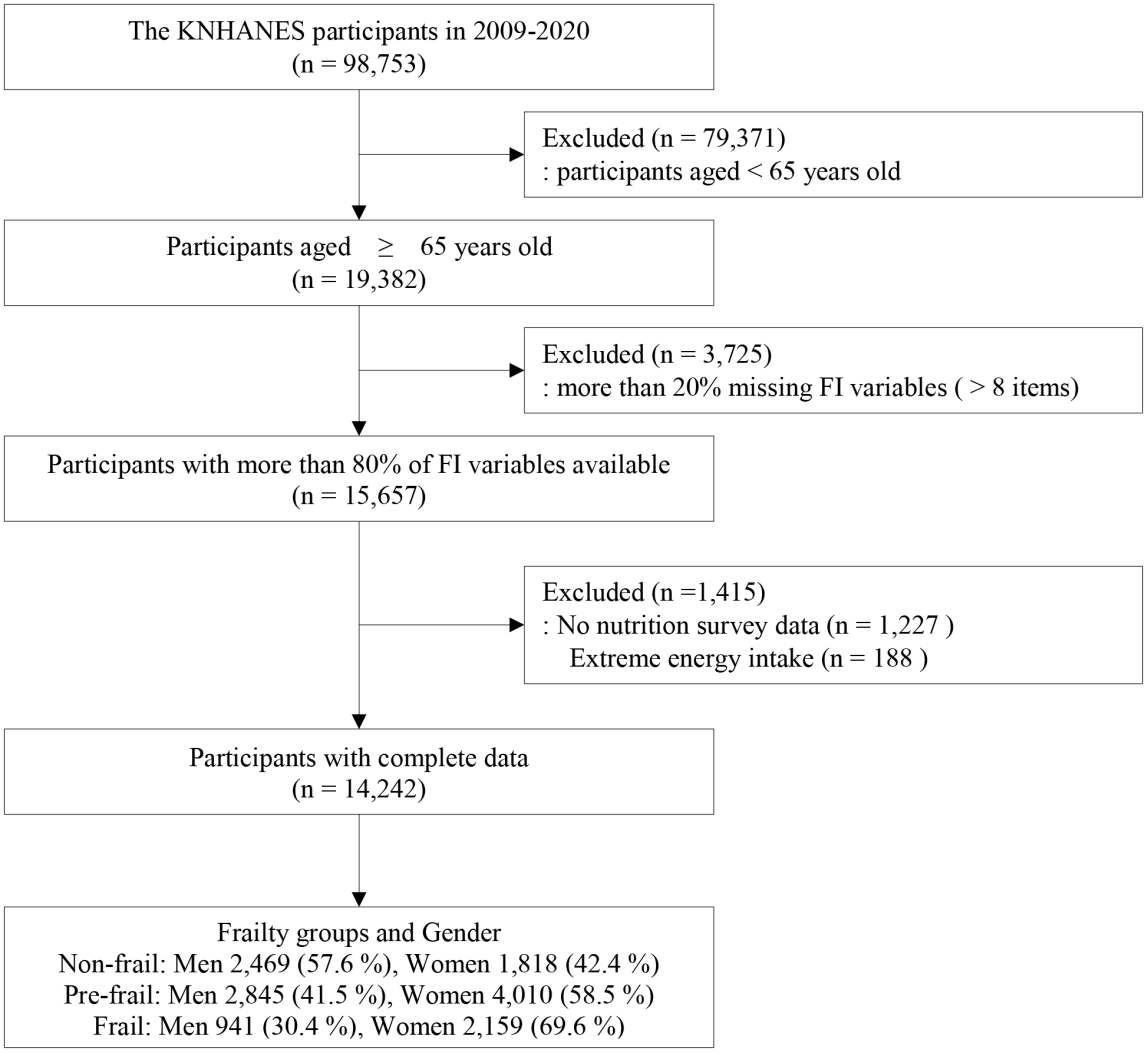
Flowchart of the study participants. KNHANES, Korea National Health and Nutrition Examination Survey; FI, frailty index.

A total of 19,382 participants aged 65 years or older were identified from the KNHANES during the study period. Of these, participants were excluded if they had more than 20% missing variables in the FI (missing FI variables > 8, n = 3,725), unavailable nutrition survey data (n = 1,277), or implausible daily energy intake (< 500 kcal or > 5,000 kcal, n = 188) [36]. After these exclusions, a total of 14,242 participants, (6,255 men and 7,987 women) were included in the final analysis (Fig 1).

### Sociodemographic and lifestyle characteristics

Sociodemographic and lifestyle characteristics were assessed using data from the KNHANES. The sociodemographic variables included age, education level, marital status, household income, and household type. Education level was categorized as middle school or below, high school, or college or above. Household income was divided into quartiles based on equalized household income: low (lowest 25%), middle-low (26%−50%), middle-high (51%−75%), and high (highest 25%). Lifestyle factors included smoking status, alcohol consumption, and meals with family. Smoking status was categorized as never smoked, quit smoked, or currently smoking. Alcohol consumption was defined as monthly drinkers (≥1/month) or non-drinkers. Meals with family were categorized based on participation in breakfast, lunch, or dinner within the previous year.

### Frailty index

Frailty reflects variability in health status among older adults and is characterized by an increased vulnerability to adverse health outcomes. In this study, frailty was assessed using a FI, a cumulative measure of health defects, ranging from 0 to 1, where higher values indicate greater frailty [13,37]. The FI was constructed using 41 variables derived from elements available in the KNHANES, following the deficit accumulation model and referencing methodologies from previous studies that developed the FI in a similar manner using national survey data [31,38–41]. To this end, variables were chosen to comprehensively represent accumulated health deficits across multiple domains, including comorbidities, physical and laboratory measures, functional limitations, symptoms, and lifestyle factors. These variables encompassed medical history and diagnose (13 items), current treatments (2 items), physical and laboratory tests (13 items), self-reported health and functional limitations (3 items), symptoms and lifestyle factors (3 items), indicators of quality of life and functional impairment (5 items), activity level (1 item), and hospitalization history (1 item). Each variable meets internationally recommended criteria: (1) relevant to health status; (2) prevalence increases with age; (3) does not saturate too early; and (4) collectively covers multiple physiological systems [37].

To ensure the stability of the frailty index (FI), it is recommended to include at least 30–40 health deficits, as the precision of FI estimates generally increases with the number of variables incorporated [37,42]. Accordingly, as the FI is typically constructed using information routinely collected in health assessments of older adults, we utilized available variables from the KNHANES dataset to develop the FI, and included 41 variables to enhance its accuracy [37]. Detailed variables and scoring criteria for the FI are provided in S1 Table. Participants were categorized into three frailty groups based on their FI score: non-frail (FI ≤ 0.15), pre-frail (0.15 < FI ≤ 0.25), and frail (FI > 0.25), in accordance with previously established thresholds shown to predict adverse health outcomes [39–41]. For instance, an FI score of 0.24, derived from 10 deficits out of 41 variables, corresponds to a pre-frail group.

### Nutrient intake assessment

Dietary intake was assessed using a 24-hour dietary recall method, with participants reporting all food and beverage consumption over a single day. To obtain accurate dietary recall data, trained dietitians conducted interviews using the multiple-pass approach in conjunction with standardized measuring guides. Nutrient intake data included total energy, macronutrients (carbohydrates, protein, fat), and micronutrients (vitamin A, thiamine, riboflavin, niacin, vitamin C, calcium, phosphorus, and iron). The percentage contributions of macronutrients to total energy intake were calculated. To assess the adequacy of nutrient intake, participants were categorized by frailty groups, and their nutrient intake levels were compared to the age and gender-specific reference values from the 2020 Korean Dietary Reference Intakes (KDRIs) [43]. In addition, the adequacy of the intake proportions of carbohydrate, protein, and fat relative to the Acceptable Macronutrient Distribution Range (AMDR) was assessed based on frailty groups. According to the 2020 KDRIs, the recommended AMDR for Korean adults is 55–65% for carbohydrate, 7–20% for protein, and 15–30% for fat. Participants whose nutrient intake was below the Estimated Average Requirement (EAR) were classified as being at risk of inadequacy, whereas those whose intake exceeded the Tolerable Upper Intake Level (UL) were assessed as being at risk of overconsumption.

### Statistical analysis

Survey weights were applied to account for the complex sampling design of the 2009–2020 KNHANES, ensuring nationally representative estimates. Categorical variables were expressed as proportions with standard error (SE) and compared using the Rao-Scott chi-square test. Continuous variables were presented as a mean ± SE and compared using ANOVA. Multivariable logistic regression analysis was performed to assess the likelihood of micronutrient intake below the EAR, with results presented as odds ratios (ORs) and 95% CIs. Linear trends across ordered frailty groups (P for trend) were evaluated using multivariable linear regression models, while gender-by-frailty groups interaction effects (P for interaction) were analyzed through interaction terms in survey regression models. All analyses were conducted using gender-stratified approaches.

Both the generalized linear model and the multivariable logistic regression analysis were adjusted for the following socio-demographic and lifestyle variables: age (in years), education level (middle school or below, high school, college or above), marital status (married/living with partner, separated/divorced, widowed, never married), household income (categorized into quartiled based on equalized household income: low, middle-low, middle-high, high), smoking status (never, past, current), and energy intake (kcal). All statistical analyses were performed using SAS software (ver. 9.4, SAS Institute Inc., Cary, NC, USA), and the level of significance was set at P < 0.05.

## Results

The baseline sociodemographic and lifestyle characteristics of the study participants, consisting of 14,242 individuals, are presented in Table 1. Women exhibited significantly lower total energy intake compared to men. Furthermore, in the frailty comparison of frailty groups, women had a lower proportion of non-frail individuals and a higher proportion of frail individuals than men. Regarding marital status, the majority of men participants were married (living with a partner, 88.9%), while women participants presented distinct patterns: 49.5% married and 45.7% widowed. Smoking status and prevalence of alcohol use were markedly lower in women compared to men. Due to significant gender-specific differences in frailty groups and sociodemographic and lifestyle characteristics, subsequent analyses were conducted using gender-stratified approaches.

**Table 1:** Basic characteristics of the study participants by gender.

Characteristics	All (n = 14,242)		Men (n = 6,255)		Women (n = 7,987)		P-value
Age (years)	72.2 ± 0.1		72.1 ± 0.1		72.4 ± 0.1		0.0128
Energy intake (kcal)	1665.5 ± 7.9		1912.3 ± 11.2		1463.0 ± 8.2		<.0001
Frailty Groups							<.0001
Non-frail	31.6	(0.5)	40.8	(0.8)	24.0	(0.6)	
Pre-frail	47.8	(0.5)	45.2	(0.8)	49.9	(0.7)	
Frail	20.6	(0.4)	14.0	(0.5)	26.1	(0.6)	
Education level							<.0001
Middle school or below	73.8	(0.6)	59.7	(0.8)	85.3	(0.6)	
High school	17.0	(0.4)	24.8	(0.6)	10.6	(0.5)	
College or above	9.2	(0.4)	15.5	(0.6)	4.0	(0.3)	
Marital status							<.0001
Married/living with partner	67.4	(0.6)	88.9	(0.5)	49.8	(0.7)	
Separated/divorced	4.0	(0.2)	4.0	(0.3)	4.1	(0.3)	
Widowed	28.1	(0.5)	6.7	(0.4)	45.7	(0.7)	
Never married	0.5	(0.1)	0.5	(0.1)	0.5	(0.1)	
Household income^a^							<.0001
Low	46.5	(0.7)	40.8	(0.8)	51.1	(0.8)	
Middle-low	26.5	(0.5)	29.3	(0.7)	24.3	(0.6)	
Middle-high	16.1	(0.5)	17.6	(0.6)	14.8	(0.5)	
High	10.9	(0.4)	12.4	(0.6)	9.7	(0.5)	
Smoking status							<.0001
Never smokers	60.2	(0.5)	19.8	(0.6)	93.3	(0.3)	
Former-smokers	29.1	(0.4)	60.1	(0.7)	3.6	(0.2)	
Current smokers	10.7	(0.3)	20.1	(0.6)	3.1	(0.3)	
Prevalence of monthly alcohol use (≥1/month)							<.0001
No	64.0	(0.5)	42.3	(0.8)	81.9	(0.5)	
Yes	36.0	(0.5)	57.7	(0.8)	18.1	(0.5)	
Meals with family in the past year							
Breakfast	64.9	(0.5)	73.9	(0.7)	57.6	(0.7)	<.0001
Lunch	56.2	(0.6)	64.7	(0.7)	49.2	(0.7)	<.0001
Dinner	68.5	(0.5)	78.6	(0.6)	60.1	(0.7)	<.0001

Continuous variables are expressed as mean ± standard error (mean ± s.e.), and categorical variables are presented as percentages with their standard error (%, s.e.).

^a^Household income was categorized into quartiles based on equalized household income: low (lowest 25%), middle-low (26%−50%), middle-high (51%−75%), and high (highest 25%).

P-values for continuous variables were calculated using F-tests, and p-values for categorical variables were calculated using the Rao-Scott Chi-Square Test.

Tables 2 and 3 showed the baseline characteristics of the study participants with gender-specific distributions of 6,255 men and 7,987 women. Participants were classified into three frailty groups: non-frail, pre-frail, and frail. Among men, 2,469 (39.5%) were classified as non-frail, 2,845 (45.5%) as pre-frail, and 941 (15.0%) as frail. In contrast, women were distributed as follows: 1,818 (22.8%) were non-frail, 4,010 (50.2%) were pre-frail, and 2,159 (27.0%) were frail. Both men and women exhibited an increase in mean age with higher levels of frailty. Similarly, the average daily energy intake decreased as frailty levels increased. Regarding smoking status, frail men had the highest proportion of former smokers (61.7%), while frail women had the highest proportion of never smokers (89.9%). Alcohol consumption patterns showed that the proportion of individuals abstaining from alcohol increased with frailty for both genders. Specifically, 57.7% of frail men and 87.2% of frail women reported no alcohol use. Likewise, the proportion of participants who did not share meals with their family over the past year increased with higher levels of frailty, regardless of the meal type.

**Table 2:** Characteristics of study participants in men according to frailty groups.

		Frailty Groups												
Characteristics		All (n = 6,255)			Non-frail (n = 2,469)			Pre-frail (n = 2,845)							Frail (n = 941)			P-value	
Age (years)	72.1 ± 0.1	71.0 ± 0.1	72.6 ± 0.1		73.8 ± 0.2		<.0001
Energy intake (kcal)	1912.3 ± 11.2			2041.9 ± 18.3	1861.3 ± 14.7		1699.3 ± 21.6		<.0001
Education level														<.0001
Middle school or below	59.7		(0.8)	50.9		(1.3)	64.4		(1.1)		69.8	(1.8)		
High school	24.8		(0.6)	27.1		(1.0)	23.9		(0.9)		21.0	(1.6)		
College or above	15.5		(0.6)	22.0		(1.1)	11.6		(0.7)		9.3	(1.2)		
Marital status														<.0001
Married/living with partner	88.9		(0.5)	91.4		(0.7)	88.3		(0.7)		83.1	(1.5)		
Separated/divorced	4.0		(0.3)	3.5		(0.5)	3.9		(0.4)		5.7	(0.9)		
Widowed	6.7		(0.4)	4.7		(0.5)	7.2		(0.6)		10.5	(1.2)		
Never married	0.5		(0.1)	0.4		(0.1)	0.5		(0.1)		0.7	(0.3)		
Household income^a^														<.0001
Low	40.8		(0.8)	32.4		(1.1)	43.4		(1.1)		57.0	(1.9)		
Middle-low	29.3		(0.7)	28.4		(1.1)	31.4		(1.1)		25.2	(1.6)		
Middle-high	17.6		(0.6)	21.3		(1.0)	16.0		(0.8)		11.5	(1.2)		
High	12.4		(0.6)	17.9		(1.0)	9.2		(0.7)		6.3	(1.0)		
Smoking status														0.0005
Never smokers	19.8		(0.6)	22.2		(1.0)	18.9		(0.9)		15.8	(1.4)		
Former-smokers	60.1		(0.7)	59.9		(1.2)	59.7		(1.1)		61.7	(1.9)		
Current smokers	20.1		(0.6)	17.9		(0.9)	21.4		(0.9)		22.4	(1.6)		
Prevalence of monthly alcohol use (≥1/month)														<.0001
No	42.3		(0.8)	36.1		(1.1)	43.1		(1.1)		57.7	(2.0)		
Yes	57.7		(0.8)	63.9		(1.1)	56.9		(1.1)		42.3	(2.0)		
Meals with family in the past year														
Breakfast	73.9		(0.7)	75.4		(1.0)	73.4		(1.0)		71.3	(1.8)		0.1103
Lunch	64.7		(0.7)	67.1		(1.1)	63.4		(1.1)		62.3	(1.8)		0.0194
Dinner	78.6		(0.6)	81.9		(0.9)	77.4		(0.9)		73.1	(1.8)		<.0001

Continuous variables are expressed as mean ± standard error (mean ± s.e.), and categorical variables are presented as percentages with their standard errors (%, s.e.).

^a^Household income was categorized into quartiles based on equalized household income: low (lowest 25%), middle-low (26%−50%), middle-high (51%−75%), and high (highest 25%).

P-values for continuous variables were calculated using F-tests, and p-values for categorical variables were calculated using the Rao-Scott Chi-Square Test.

**Table 3:** Characteristics of study participants in women according to frailty groups.

	Frailty Groups								
Characteristics	All (n = 7,987)		Non-frail (n = 1,818)		Pre-frail (n = 4,010)		Frail (n = 2,159)		P-value
Age (years)	72.4 ± 0.1		70.3 ± 0.1		72.5 ± 0.1		74.0 ± 0.1		<.0001
Energy intake (kcal)	1463.0 ± 8.2		1599.2 ± 16.4		1459.9 ± 11.4		1343.4 ± 13.7		<.0001
Education level									<.0001
Middle school or below	85.3	(0.0)	74.4	(1.4)	86.0	(0.7)	94.2	(0.6)	
High school	10.6	(0.5)	16.7	(1.1)	10.9	(0.7)	4.4	(0.5)	
College or above	4.0	(0.3)	8.9	(0.9)	3.1	(0.3)	1.4	(0.3)	
Marital status									<.0001
Married/living with partner	49.8	(0.7)	61.3	(1.4)	49.5	(1.0)	39.7	(1.3)	
Separated/divorced	4.1	(0.3)	3.8	(0.6)	4.2	(0.4)	4.0	(0.5)	
Widowed	45.7	(0.7)	34.4	(1.4)	45.8	(1.0)	55.7	(1.3)	
Never married	0.5	(0.1)	0.4	(0.1)	0.6	(0.1)	0.6	(0.2)	
Household income^a^									<.0001
Low	51.1	(0.8)	41.3	(1.4)	50.4	(1.0)	61.6	(1.3)	
Middle-low	24.3	(0.6)	27.6	(1.2)	24.3	(0.8)	21.2	(1.1)	
Middle-high	14.8	(0.5)	18.2	(1.2)	15.3	(0.7)	10.8	(0.9)	
High	9.7	(0.5)	12.9	(1.0)	10.0	(0.6)	6.3	(0.6)	
Smoking status									<.0001
Never smokers	93.3	(0.3)	95.3	(0.6)	94.1	(0.5)	89.9	(0.8)	
Former-smokers	3.6	(0.2)	2.2	(0.4)	3.1	(0.3)	5.8	(0.6)	
Current smokers	3.1	(0.3)	2.5	(0.5)	2.7	(0.3)	4.3	(0.6)	
Prevalence of monthly alcohol use (≥1/month)									<.0001
No	81.9	(0.5)	76.8	(1.2)	81.6	(0.7)	87.2	(0.9)	
Yes	18.1	(0.5)	23.2	(1.2)	18.4	(0.7)	12.8	(0.9)	
Meals with family in the past year									
Breakfast	57.6	(0.7)	64.0	(1.3)	57.7	(0.9)	51.5	(1.3)	<.0001
Lunch	49.2	(0.7)	51.9	(1.4)	50.5	(1.0)	44.0	(1.3)	<.0001
Dinner	60.1	(0.7)	67.5	(1.3)	60.8	(1.0)	52.1	(1.3)	<.0001

Continuous variables are expressed as mean ± standard error (mean ± s.e.), and categorical variables are presented as percentages with their standard errors (%, s.e.).

^a^Household income was categorized into quartiles based on equalized household income: low (lowest 25%), middle-low (26%−50%), middle-high (51%−75%), and high (highest 25%).

P-values for continuous variables were calculated using F tests, and p-values for categorical variables were calculated using the Rao-Scott Chi-Square Test.

Tables 4 and 5 provide the daily nutrient intake of study participants categorized by frailty groups separately for men and women. For men, riboflavin intake was 1.26 mg (95% CI: 1.22–1.30) in the non-frail group, 1.19 mg (95% CI: 1.16–1.22) in the pre-frail group, and 1.21 mg (95% CI: 1.16–1.25) in the frail group. The mean values did not exhibit a consistent directional trend across frailty groups. However, multivariable linear regression analysis revealed a statistically significant association between frailty levels and riboflavin intake (P for trend = 0.0012). In contrast, no significant trends were observed for other vitamins, macronutrients, and minerals (P for trend > 0.05). For women, carbohydrate intake increased significantly with higher frailty levels (non-frail: 260.11 g; pre-frail: 263.93 g; frail: 265.55 g; P for trend = 0.005), while fat intake decreased significantly (non-frail: 25.02 g; pre-frail: 23.71 g; frail: 23.12 g; P for trend = 0.0032). Riboflavin intake, similar to that observed in men, decreased significantly across groups (non-frail: 1.01 mg; pre-frail: 1.00 mg; frail: 0.99 mg; P for trend = 0.0062). Furthermore, the analysis revealed gender- specific differences in nutrient intake according to frailty groups. Frail women exhibited a significantly higher LS-mean carbohydrate ratio compared to frail men, while frail men showed a higher fat ratio than frail women (interaction p = 0.0133 for carbohydrate ratio, and p = 0.0024 for fat ratio). Significant gender differences were also observed in iron intake, with frail men exhibiting higher levels compared to frail women (interaction p = 0.0149).

**Table 4:** Least-squares means (95% confidence intervals) of daily nutrient intake of study participants in men according to frailty groups.

	LS-means^a^ (95% CIs^b^) according to Frailty Groups			P-value	
Nutrients	Non-frail (n = 2,469)	Pre-frail (n = 2,845)	Frail (n = 941)	Trend	Interaction
Carbohydrate (g)	326.07 (322.42 - 329.72)	328.59 (324.97 - 332.22)	325.57 (320.37 - 330.76)	0.3325	0.6136
Protein (g)	65.35 (64.24 - 66.46)	64.27 (63.15 - 65.40)	64.56 (62.98 - 66.13)	0.1705	0.8458
Fat (g)	31.78 (30.69 - 32.86)	31.26 (30.25 - 32.27)	32.05 (30.67 - 33.43)	0.4360	0.0435
Macronutrient ratio					
Carbohydrate ratio (%)	71.20 (70.61 - 71.79)	71.59 (71.00 - 72.18)	71.17 (70.32 - 72.01)	0.3649	0.0133
Protein ratio (%)	14.07 (13.83 - 14.31)	13.82 (13.57 - 14.07)	13.95 (13.53 - 14.38)	0.1059	0.7388
Fat ratio (%)	14.76 (14.30 - 15.21)	14.60 (14.15 - 15.05)	14.78 (14.15 - 15.41)	0.7636	0.0024
Vitamins					
Vitamin A (μg RAE^c^)	360.71 (326.48 - 394.95)	362.23 (338.15 - 386.30)	350.97 (317.19 - 384.74)	0.7745	0.9031
Thiamine (mg)	1.42 (1.38 - 1.46)	1.40 (1.37 - 1.44)	1.38 (1.34 - 1.43)	0.4044	0.3026
Riboflavin (mg)	1.26 (1.22 - 1.30)	1.19 (1.16 - 1.22)	1.21 (1.16 - 1.25)	0.0012	0.2349
Niacin (mg NE^d^)	13.43 (13.11 - 13.76)	13.30 (13.00 - 13.60)	13.17 (12.74 - 13.60)	0.5362	0.3335
Vitamin C (mg)	78.23 (73.50 - 82.96)	81.67 (76.67 - 86.67)	76.75 (70.67 - 82.84)	0.2212	0.0934
Minerals					
Calcium (mg)	504.65 (486.45 - 522.86)	488.87 (470.75 - 506.98)	498.84 (473.32 - 524.35)	0.2810	0.6351
Phosphorus (mg)	1082.55 (1065.10 - 1099.99)	1067.35 (1050.65 - 1084.06)	1063.94 (1040.36 - 1087.52)	0.1952	0.7282
Sodium (mg)	3771.55 (3642.43 - 3900.67)	3874.51 (3740.29 - 4008.72)	3809.99 (3640.64 - 3979.34)	0.3423	0.7736
Potassium (mg)	2970.10 (2903.57 - 3036.63)	2900.64 (2837.38 - 2963.91)	2888.07 (2806.36 - 2969.78)	0.0940	0.1992
Iron (mg)	14.11 (13.50 - 14.72)	13.98 (13.47 - 14.49)	13.57 (12.93 - 14.22)	0.3008	0.0149

^a^LS-means = least-squares means.

^b^CIs = confidence intervals.

^c^RAE = retinol activity equivalent.

^d^NE = niacin equivalent.

Models were adjusted for age(year), education(middle school or below, high school, college or above), marital status(married/living with partner, separated/divorced, widowed, never married), household income(low, middle-low, middle-high, high), smoking status(never, past, ever), and energy intake(kcal).

P for trend values were calculated using multivariable linear regression models to evaluate the linear trend across ordered categories of frailty groups.

P for interaction values were obtained from the gender by frailty category interaction term in the survey regression model.

**Table 5:** Least-squares means (95% confidence intervals) of daily nutritional intake of study participants in women according to Frailty Groups.

	LS-means^a^ (95% CIs^b^) according to Frailty Groups			
Nutrients	Non-frail (n = 1,818)	Pre-frail (n = 4,010)	Frail (n = 2,159)	P for trend
Carbohydrate (g)	260.11 (257.15 - 263.08)	263.93 (261.58 - 266.29)	265.55 (262.75 - 268.35)	0.0050
Protein (g)	48.48 (47.56 - 49.40)	47.71 (46.98 - 48.44)	47.84 (46.93 - 48.75)	0.2646
Fat (g)	25.02 (24.00 - 26.04)	23.71 (22.91 - 24.51)	23.12 (22.17 - 24.07)	0.0032
Macronutrient ratio				
Carbohydrate ratio (%)	71.98 (71.29 - 72.67)	72.87 (72.30 - 73.44)	73.34 (72.66 - 74.03)	0.0018
Protein ratio (%)	13.19 (12.94 - 13.43)	13.01 (12.81 - 13.20)	12.98 (12.73 - 13.23)	0.2560
Fat ratio (%)	14.83 (14.28 - 15.38)	14.12 (13.66 - 14.58)	13.68 (13.14 - 14.22)	0.0006
Vitamins				
Vitamin A (μg RAE^c^)	294.79 (272.04 - 317.54)	312.28 (282.95 - 341.60)	313.62 (285.20 - 342.03)	0.5358
Thiamine (mg)	1.04 (1.01 - 1.07)	1.06 (1.04 - 1.09)	1.03 (1.00 - 1.06)	0.0468
Riboflavin (mg)	1.01 (0.98 - 1.05)	0.97 (0.94 - 1.00)	0.95 (0.92 - 0.98)	0.0064
Niacin (mg NE^r^)	10.01 (9.73 - 10.29)	10.05 (9.85 - 10.25)	10.19 (9.92 - 10.45)	0.4701
Vitamin C (mg)	78.30 (71.31 - 85.29)	76.24 (70.32 - 82.17)	74.64 (68.80 - 80.48)	0.5199
Minerals				
Calcium (mg)	402.57 (386.63 - 418.52)	401.07 (387.64 - 414.50)	399.71 (379.07 - 420.34)	0.9706
Phosphorus (mg)	829.39 (814.92 - 843.87)	825.99 (814.62 - 837.37)	826.56 (813.52 - 839.61)	0.9032
Sodium (mg)	2743.55 (2625.29 - 2861.80)	2861.57 (2763.43 - 2959.72)	2848.05 (2728.43 - 2967.66)	0.1101
Potassium (mg)	2390.61 (2328.61 - 2452.60)	2391.86 (2339.49 - 2444.22)	2345.20 (2285.32 - 2405.07)	0.2394
Iron (mg)	10.31 (9.82 - 10.79)	10.94 (10.45 - 11.44)	11.80 (11.04 - 12.56)	0.0046

^a^LS-means = least-squares means.

^b^CIs = confidence intervals.

^c^RAE = retinol activity equivalent.

^d^NE = niacin equivalent.

Models were adjusted for age(year), education(middle school or below, high school, college or above), marital status(married/living with partner, separated/divorced, widowed, never married), household income(low, middle-low, middle-high, high), smoking status(never, past, ever), and energy intake(kcal).

P for trend values were calculated using multivariable linear regression models to evaluate the linear trend across ordered categories of frailty Groups.

**Fig 2:**
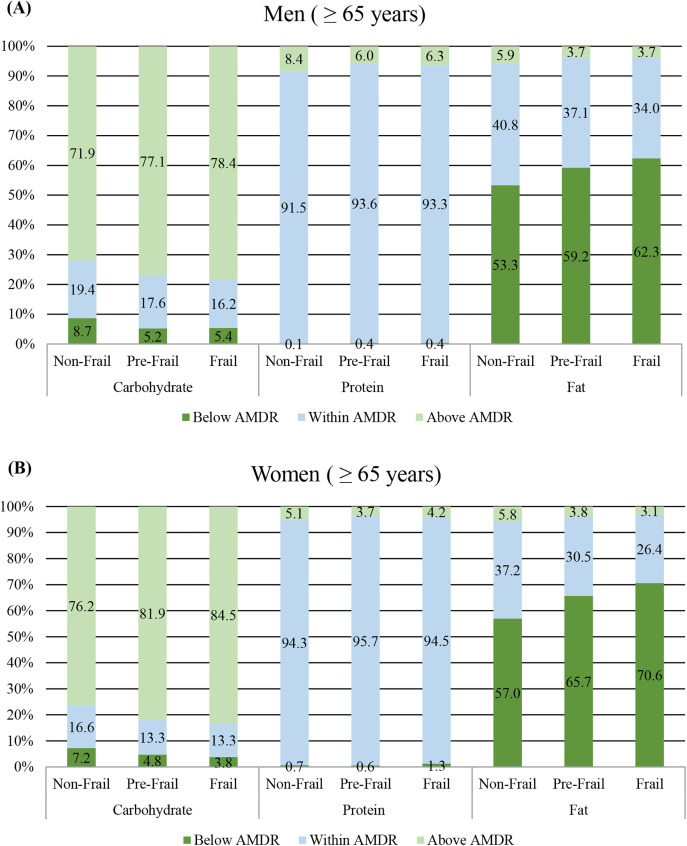
Percentage of macronutrient intake relative to the acceptable macronutrient distribution range (AMDR) by frailty groups.

The percentage of frailty groups (non-frail, pre-frail, and frail) with macronutrient intake below, within, and above the AMDR is summarized in Fig 2. For carbohydrate intake, the proportion of individuals above the AMDR increased slightly as frailty groups progressed, reaching 78.4% in frail men and 84.5% in frail women. Protein intake was consistently within the AMDR for the majority of participants across all frailty groups in both men and women. Conversely, fat intake showed a high proportion of participants falling below the AMDR across all frailty groups. The frail group exhibited a higher percentage of individuals below the AMDR for fat intake (men: 62.3%, women: 70.6%) compared to the non-frail group (men: 53.3%, women: 57.0%).

**Fig 3:**
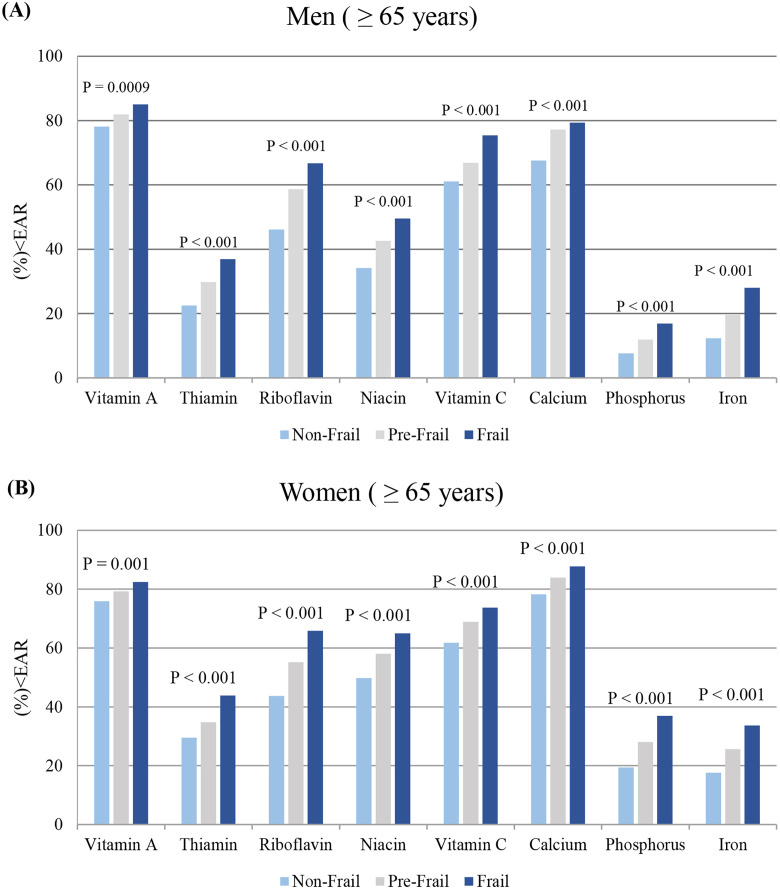
Percentage of participants with micronutrient intakes below the estimated average requirement (EAR) by frailty groups.

Micronutrient intake was also evaluated according to age and gender-specific EAR and UL. Fig 3 presents the percentage of participants aged 65 years or older whose intakes were below the EAR, categorized by frailty groups (non-frail, pre-frail, and frail) for men (A) and women (B). Across all micronutrients analyzed, the frail group consistently exhibited a higher proportion of intakes below the EAR compared to the non-frail group in both men and women. In men, 85.1% of frail individuals had intakes below the EAR for vitamin A, as detailed in S2 Table. Similarly, in women, over 80% of frail individuals had intakes below the EAR for both vitamin A (82.5%) and calcium (87.7%), as shown in S3 Table. In cases of excessive intake above the UL for micronutrients, the proportions were as follows: vitamin A (0.1% in men, 0.3% in women), calcium (0.4% in men, 0.2% in women), and iron (0.7% in men, 1.3% in women), while both vitamin C and phosphorus were 0% in both genders (S4 Table).

The association between frailty groups and the likelihood of micronutrient intakes below the EAR was analyzed for men (Table 6) and women (Table 7). Among men, a significant trend was observed in riboflavin intake below the EAR across frailty groups (p for trend = 0.0250), although the odds ratio for the frail group (ORs: 1.23, 95% CI: 0.99–1.54) did not reach statistical significance. In contrast, iron intake below the EAR showed both a significant trend (p for trend = 0.0018) and odds in the frail group (ORs: 1.49, 95% CI: 1.15–1.94). For women, riboflavin intake below the EAR was significantly associated with frailty groups (ORs: 1.45, 95% CI: 1.20–1.74), with a clear trend observed across groups (p for trend < 0.0001). Additionally, thiamin intake below the EAR demonstrated a significant trend across the frailty group (p for trend = 0.0365), although the odds ratio for the frail group indicated a borderline association (ORs: 1.22, 95% CI: 1.00–1.48). No significant interaction effects between gender and frailty status were observed for any micronutrient intake deficiencies (interaction p > 0.05 for all).

**Table 6:** Odds ratios and 95% confidence intervals of micronutrient intakes below the estimated average requirement (EAR) in men according to frailty groups.

	Frailty Groups			P-value	
Micronutrient intakes below the EAR	Non-frail (n = 2,496)	Pre-frail (n = 2,845)	Frail (n = 941)	Trend	Interaction
Vitamin A	1	0.96 (0.81 - 1.14)	0.98 (0.75 - 1.26)	0.9433	0.9995
Thiamin	1	1.07 (0.90 - 1.26)	1.06 (0.84 - 1.34)	0.5463	0.1990
Riboflavin	1	1.19 (1.02 - 1.39)	1.23 (0.99 - 1.54)	0.0250	0.2001
Vitamin C	1	0.97 (0.84 - 1.12)	1.23 (1.00 - 1.51)	0.1900	0.2048
Niacin	1	1.00 (0.85 - 1.18)	0.89 (0.70 - 1.14)	0.4525	0.4393
Calcium	1	1.21 (1.03 - 1.43)	1.05 (0.82 - 1.35)	0.2153	0.3744
Phosphorus	1	1.06 (0.80 - 1.40)	1.01 (0.72 - 1.42)	0.9210	0.8744
Iron	1	1.29 (1.05 - 1.58)	1.49 (1.15 - 1.94)	0.0018	0.6546

Models were adjusted for age(year), education(middle school or below, high school, college or above), marital status(married/living with partner, separated/divorced, widowed, never married), household income(low, middle-low, middle-high, high), smoking status(never, past, ever), and energy intake(kcal).

A multivariable logistic regression analysis was conducted to determine the association between micronutrient intakes below the EAR and frailty groups, and P for trend was used to evaluate the linear trend across frailty groups.

P for interaction values were obtained from the gender by frailty category interaction term in the survey regression model.

**Table 7:** Odds ratios and 95% confidence intervals of micronutrient intakes below the estimated average requirement (EAR) in women according to frailty groups.

	Frailty Groups			
Micronutrient intakes below the EAR	Non-frail (n = 1,818)	Pre-frail (n = 4,010)	Frail (n = 2,159)	P for trend
Vitamin A	1	0.90 (0.76 - 1.06)	0.86 (0.70 - 1.06)	0.4475
Thiamin	1	0.97 (0.82 - 1.16)	1.22 (1.00 - 1.48)	0.0365
Riboflavin	1	1.18 (1.01 - 1.38)	1.45 (1.20 - 1.74)	<.0001
Vitamin C	1	1.03 (0.88 - 1.20)	1.02 (0.85 - 1.22)	0.8260
Niacin	1	0.97 (0.81 - 1.16)	0.98 (0.79 - 1.21)	0.8469
Calcium	1	1.06 (0.88 - 1.29)	1.12 (0.87 - 1.43)	0.3800
Phosphorus	1	1.01 (0.82 - 1.26)	1.02 (0.79 - 1.30)	0.9042
Iron	1	1.15 (0.94 - 1.41)	1.22 (0.98 - 1.53)	0.0889

Models were adjusted for age(year), education(middle school or below, high school, college or above), marital status(married/living with partner, separated/divorced, widowed, never married), household income(low, middle-low, middle-high, high), smoking status(never, past, ever), and energy intake(kcal).

A multivariable logistic regression analysis was conducted to determine the association between micronutrient intakes below the EAR and frailty groups, and P for trend was used to evaluate the linear trend across frailty groups.

## Discussion

This study aimed to investigate the prevalence of frailty among older adults in South Korea and to examine the differences in nutritional status according to frailty. Utilizing a multidimensional frailty index, the study explored differences in nutrition, demographic characteristics, health behaviors, and social relationships by gender and frailty group to identify key characteristics and issues associated with frailty. Based on these findings, the study sought to provide foundational evidence for the development of targeted dietary and nutritional policies and interventions for the prevention of frailty.

The results indicated that individuals in the frail group exhibited more disadvantaged demographic characteristics, health behaviors, social relationships, and nutritional status compared to the non-frail group. The frail group had lower education and income levels, higher smoking experience rates, and lower rates of shared meals. These findings also suggest that individuals in the frail group may have limited access to social and economic resources, particularly with respect to opportunities for social interaction. As discussed in detail below, such reduced opportunities for social eating may have broad implications for nutritional status and mental health among older adults. The following characteristics are presented in sequence, focusing on nutrition, social relationships, and health behaviors.

First, in terms of nutrition, a higher proportion of individuals in the frail group consumed carbohydrates above the upper limit of the AMDR and fat below the lower limit. These findings are consistent with previous research indicating that replacing energy intake from fat with carbohydrates may significantly increase the risk of frailty, and that higher carbohydrate intake is positively associated with frailty risk [44,45]. In a clinical study involving adults aged 21–50 years, high carbohydrate intake was shown to impair insulin sensitivity and promote the accumulation of abdominal and intermuscular fat, thereby deteriorating metabolic health [46]. These changes were further associated with reduced muscle mass and increased insulin secretion. Such metabolic mechanisms are likely to operate similarly across different age groups. Indeed, findings from the Health ABC study, which focused on older adults, demonstrated that dietary patterns characterized by high intakes of refined carbohydrates, sugars, and high-fat dairy products were associated with increased insulin resistance and heightened inflammatory responses, ultimately worsening metabolic health in the elderly population [47]. This highlights the importance of addressing macronutrient imbalance as a nutritional risk factor for frailty.

Our study confirmed that frailty levels are differentially associated with deficiencies in micronutrients such as riboflavin, thiamin, and iron, depending on gender. In particular, inadequate riboflavin intake was associated with an increased risk of frailty in both men and women, which is consistent with previous findings showing significantly lower mean riboflavin intake in frail individuals [48]. However, a systematic review found that potential thiamin and riboflavin deficiencies among older adults are not considered to have major public health implications [49]. This discrepancy may stem from differences in outcome variables: prior studies primarily assessed clinical deficiency symptoms such as cheilosis, glossitis [50], whereas our study employed a multidimensional frailty index to capture the cumulative effects of long-term nutritional imbalances [30,51].

For riboflavin, impaired coenzyme production—which is critical for energy metabolism—may contribute to subclinical muscle mass reduction and fatigue, potentially exacerbating frailty without overt deficiency signs [52,53]. This is because riboflavin serves as a precursor for flavin adenine dinucleotide and flavin mononucleotide, both of which are essential cofactors in mitochondrial oxidative phosphorylation and fatty acid beta-oxidation, supporting intracellular ATP production [50]. Consequently, riboflavin deficiency may result in diminished mitochondrial function, impaired skeletal muscle energy metabolism, and reduced antioxidant defense due to decreased glutathione reductase activity, which can collectively manifest as muscle weakness, increased fatigue, and heightened vulnerability to frailty—even in the absence of overt clinical signs of deficiency [50–54].

Additionally, among frail men, inadequate iron intake was associated with an increased risk of frailty exclusively in men. The Concord Health and Ageing in Men Project, a study conducted in Australia, reported that higher total dietary iron intake was associated with a reduced incidence of frailty among older men, similarly suggesting a potential link between iron deficiency and the development of frailty, which is consistent with the findings of the present study [55]. Iron plays a critical role in several physiological functions, including oxygen transport, mitochondrial energy production, and skeletal muscle metabolism, and iron deficiency can lead to iron-deficiency anemia, which reduces tissue oxygen delivery and contributes to decreased exercise tolerance, muscle weakness, and increased fatigue—symptoms commonly observed in frailty [56]. Moreover, iron deficiency can impair mitochondrial function, reduce muscle energy production, and promote sarcopenia, thereby exacerbating frailty even before the onset of anemia [56,57]. Although older men generally possess greater skeletal muscle mass, which may render them more susceptible to functional decline under conditions of iron imbalance, anemia remains a critical factor associated with frailty in both genders [58]. Therefore, comprehensive assessment and management of iron deficiency and anemia should be prioritized in the elderly population, regardless of gender. This aligns with previous findings where multivitamin/mineral supplement interventions reduced frailty risk in low-income populations, highlighting the need for community-based, tailored nutritional support programs [59]. Especially for older men, tailored nutritional interventions are required, such as promoting the intake of iron-rich foods (e.g., lean meats, liver, legumes) and considering supplementation strategies that account for absorption efficiency. In addition, comprehensive nutritional assessments and dietary management are essential, given the common issues of overall protein inadequacy, reduced appetite, and dental problems. Additionally, further studies are required to elucidate the long-term impact of micronutrient deficiencies on frailty progression.

Second, in terms of social relationships, the frail group had a significantly lower rate of sharing meals with others. Shared meals are not merely dietary behavior but also reflect emotional support and structured daily routines, which are critical components for healthy aging. Previous studies have demonstrated that social isolation is associated with increased depressive symptoms and reduced dietary quality among older adults [60,61]. These findings suggest that individuals in the frail group may have limited access to social and economic resources, particularly with respect to opportunities for social interaction. The absence of regular social engagement has been associated with suboptimal dietary behaviors and may further contribute to poor mental health outcomes. According to previous studies, individuals who eat with others are more likely to select healthier food options and consume more nutritionally balanced meals, a pattern that may be explained by the influence of perceived social norms and behavioral modeling mechanisms [62,63]. Moreover, healthy eating behaviors have been shown to spread through social networks, and dietary norms established within a social group can influence the food choices of other socially connected individuals [64,65]. In older adults, social isolation has been associated with negative dietary outcomes, such as reduced appetite, irregular eating patterns, and insufficient nutrient intake; furthermore, among community-dwelling older individuals living alone, limited social interaction may further increase the risk of undernutrition [66]. The Korean Frailty and Aging Cohort Study examined the impact of changes in eating companionship on frailty progression among older adults in Korea [67]. This highlights the importance of community-based communal meal programs and policies that promote social connections, led by local community service centers. Such initiatives can reduce social isolation among older adults and foster social interaction through shared meals. In turn, they help maintain healthy dietary habits and support mental health, making them an effective strategy for frailty prevention.

Third, from a health behavioral perspective, both frail men and women exhibited higher rates of past and current smoking. Particularly among men, the current smoking rate was notably higher in the frail group, suggesting that gender-specific smoking cessation interventions are necessary. Conversely, a higher proportion of lifelong non-smokers was observed among the non-frail group, implying that non-smoking may act as a protective factor against frailty. Notably, a study utilizing data from the Chinese Longitudinal Healthy Longevity Survey demonstrated that current smoking was significantly associated with an elevated risk of frailty among older men, emphasizing the adverse impact of smoking on frailty status in this population [68]. Unlike programs targeting younger adults, smoking cessation interventions for older adults should be designed with careful consideration of their psychological resistance and established lifestyle patterns. Evidence suggests that tailored counseling programs are more effective than generic interventions [69]. Accordingly, customized approaches—such as peer-led smoking cessation education and nicotine replacement therapy—are particularly needed for this population, especially among older men. Regarding alcohol consumption, the frail group showed a lower prevalence of drinking experience. Although previous studies have generally suggested that alcohol consumption negatively affects health [70,71], the relationship between alcohol intake and frailty remains inconclusive [72,73]. In the present study, a higher prevalence of drinking experience was observed among the non-frail group. This finding implies that alcohol consumption might not merely represent a health risk behavior but could also serve as an opportunity for social engagement [73]. These findings highlight the need for further research into the association between social relationships and the degree of frailty in later life, as well as the social role and significance of alcohol consumption as a means of maintaining interpersonal connections.

Finally, looking at the overall differences in aging between men and women, women had a higher proportion of frailty than men. Although women exhibited more favorable health behaviors, such as lower rates of smoking and alcohol consumption compared to men, the proportion of frailty was higher among women. This finding contrasts with previous studies reporting a negative association between health behaviors and frailty [74,75], suggesting that frailty in women may be more strongly influenced by demographic and relational factors rather than health behaviors alone. In particular, older women had higher rates of living alone, lower income levels, and fewer shared meals with family members, highlighting that structural and emotional support may be associated with frailty. Emotional support and social relationships have been shown to influence the onset of diseases, functional decline, and depression in older adults [76,77]. Therefore, frailty prevention and management strategies for older women should extend beyond promoting healthy behaviors to include structural interventions that strengthen social networks. For instance, fostering participation in communal meal programs, local community-based services, and emotional support initiatives—such as conversation groups and peer counseling—can play a pivotal role in improving well-being among older women.

Furthermore, research indicates that men and women differ significantly in health behaviors, relationship patterns, and access to social resources. As such, gender-specific approaches are essential in the design of frailty prevention interventions. For example, strategies targeting smoking cessation and nutritional support may be more effective for men, while those emphasizing emotional support and social participation may better serve women. Gender-tailored interventions not only address the limitations of one-size-fits-all models but also contribute to more efficient and equitable resource allocation in policy development.

This study has some limitations. First, as it was based on a cross-sectional design, it was only possible to identify associations between frailty and related factors, and causal relationships could not be determined. Nevertheless, the KNHANES provides reliable, population-based estimates of the health and nutritional status of older adults in Korea, due to its nationally representative sampling design and standardized data collection methods. Future longitudinal studies are needed to elucidate the causal effects of demographic, behavioral, relational, and nutritional factors on frailty, with particular attention to gender differences. Second, the measurement of alcohol consumption in this study was limited and coarse; therefore, future research should adopt more precise measures, including drinking frequency, quantity, and contextual factors, and should incorporate qualitative approaches to understanding older adults’ perceptions of alcohol use. Third, although dietary intake data collected using the 24-hour recall method reflect an individual’s consumption over the previous day and may not fully represent habitual dietary patterns or actual nutrient intake, this method is widely utilized as a standard approach to estimate average nutrient intakes at the population level [78]. Lastly, as this study focused on older adults in Korea, the findings may not be directly generalizable to older populations in different cultural or dietary contexts. Nevertheless, the associations among frailty, nutritional status, social relationships, and health behaviors have been consistently observed across various countries. The key contribution of this study lies not only in reaffirming these associations but also in underscoring the importance of analyzing older adults as heterogeneous subgroups—particularly by gender and frailty severity—rather than treating them as a uniform population. This approach highlights the necessity of developing tailored interventions that reflect the distinct characteristics of each subgroup. Accordingly, future research in other countries should seek to identify and classify frailty subtypes within their own sociocultural frameworks to inform the design of more effective and contextually relevant interventions.

## Conclusions

This study confirmed that, despite demonstrating relatively healthy lifestyle behaviors, older women had higher levels of frailty, suggesting that frailty among women is closely associated with demographic and social relational vulnerabilities. Moreover, regardless of gender, individuals in the frail group exhibited compounded vulnerabilities across demographic, behavioral, relational, and nutritional domains, providing foundational insights for the development of targeted policies and community-based programs.

By applying a multidimensional frailty index, this study captured not only physical health risks but also behavioral patterns and social relational structures. Of particular note, alcohol consumption exhibited a different pattern compared to smoking, underscoring the need for programs that promote healthy social interactions among older adults. Furthermore, this study highlighted that differences in nutrient intake patterns by gender and the presence of micronutrient deficiencies are significantly associated with frailty. Consequently, future frailty prevention policies should incorporate gender-specific nutritional interventions. Collectively, these findings provide important groundwork for designing integrated strategies to improve the quality of life among older adults.

## Supporting information

S1 Table The 41-item frailty index. (DOCX)

S2 Table Proportions of participants with intakes below the estimated average requirement (EAR) according to frailty groups in men. (DOCX)

S3 Table Proportions of participants with intakes below the estimated average requirement (EAR) according to frailty groups in women. (DOCX)

S4 Table Proportions of participants with intakes below the tolerable upper intake (UL) according to frailty groups. (DOCX)

## Abbreviations

**AMDR:**: acceptable macronutrient distribution range
CIs: confidence intervals
**EAR:**: estimated average requirement
**FI**: frailty index
**KNHANES**: Korean National Health and Nutrition Examination Survey
ORs: odds ratios
**SE**: standard error.
