# Supplementary material for: Frailty and nutritional inadequacy in older Korean adults: A gender-stratified analysis using National Survey Data
Source: PLoS One. 2025 Oct 27;20(10):e0333620. doi: 10.1371/journal.pone.0333620 (PMC12558530; doi:10.1371/journal.pone.0333620)
Supplement: S1 Table — (DOCX) [file pone.0333620.s001.docx]

S1 Table. The 41-item frailty index.

| Frailty index variables | Scoring |
| --- | --- |
| **Medical history and diagnoses** |  |
| 1. Ever diagnosed with stroke | 0= No; 1=Yes |
| 2. Ever diagnosed with myocardial infarction (MI) |  |
| 3. Ever diagnosed with angina pectoris |  |
| 4. Ever diagnosed with cancer (other than skin cancer) |  |
| 5. Ever diagnosed with rheumatic arthritis |  |
| 6. Ever diagnosed with osteoarthritis |  |
| 7. Ever diagnosed with thyroid illness |  |
| 8. Ever diagnosed with hepatitis B |  |
| 9. Hypertension | 0 = No; 1 = Yes (SBP ≥140 mmHg or DBP ≥90 or taking anti-hypertensive) |
| 10. Hypotension | 0=No; 1=Yes (SBP<90 mmHg or DBP<60) |
| 11. Hypercholesterolemia | 0=No; 1= Yes (≥240 mg/dL after fasting 8 or more hours, or taking lipid control medications) |
| 12. Hypertriglyceridemia | 0=No; 1=High (≥200 mg/dL after fasting 12 or more hours) |
| 13. Diabetes | 0 = No; 1 = Yes (Glucose ≥126 mg/dL after fasting 8 or more hours, or diagnosed with diabetes by a doctor, or taking glucose-lowering medication or administering insulin) |
| **Current treatments** |  |
| 1. Current treatment of depression | 0= No; 1=Yes |
| 2. Current treatment of asthma |  |
| **Physical and laboratory tests** |  |
| 1. Anemia | 0=No; 1=Yes (M: Hemoglobin [g/dL] <13, W: <12) |
| 2. Low weight | 0=Not low; 1=Low (BMI <18.5 kg/m2) |
| 3. Obese | 0=Not obese (<23); 0.5=Slightly obese (≥25 and <30);0.25=Borderline obese (≥23 and <25); 0.75=Obese (≥30 and <35); 1=Most obese (BMI ≥35 kg/m2) |
| 4. Irregular heart rate | 0=No; 1=Yes |
| 5. Creatinine clearance (CrCl) | 0=Not low; 1=Low (Cockcroft-Gault CrCl <60 mL/min) |
| 6. Hematocrit | 0=Not low; 1=Low (≤24%) |
| 7. White blood cell count (WBC) | 0=4~10 thous/uL (inclusive); else 1 |
| 8. Red blood cell count (RBC) | 0=M: 4.2~6.3 million cells/uL, F: 4.0~5.4 (inclusive); else 1 |
| 9. Platelet count | 0=150~450 thous/uL (inclusive); else 1 |
| 10. Hemoglobin A1c (HbA1c) | 0 = Not high (≤5.6%); 0.5 = Borderline (>5.6% and <6.5%); 1 = Uncontrolled (≥6.5%) |
| 11. Alanine transaminase (ALT) | 0 = ≤ULN; 0.5 = >ULN, ≤2x ULN; 1 = >2x ULN  (ULN, M: >33 IU/L, W: >25 IU/L) |
| 12. Aspartate transaminase (AST) | 0 = <ULN; 0.5 = ≥ULN, <2x ULN; 1 = ≥2x ULN (ULN, 20 IU/L) |
| 13. High-density lipoprotein (HDL) | 0=No; 1=Low (<40 mg/dL after fasting 8 or more hours) |
| **Self-reported health and functional limitations** |  |
| 1. Poor self-reported health | 0 = Very good-good; 0.5 = Normal;1 = Bad-very bad |
| 2. Any current restrictions in daily living or social activity  due to illness, physical or mental disorder | 0= No; 1=Yes |
| 3. Any stays in bed almost all day in the last month |  |
| **Symptoms and lifestyle factors** |  |
| 1. Unintentional weight loss (more than 3 kg) within 1 year | 0= No; 1=Yes |
| 2. Problems with chewing (survey) | 0 = Not at all/Not really/Okay; 0.5 = Uncomfortable;  1 = Very uncomfortable |
| 3. Problems with talking (survey) |  |
| **Indicators of quality of life and functional impairment** |  |
| 1. EQ5D: Difficulty walking | 0= No; 0.5=A little; 1=Severe |
| 2. EQ5D: Difficulty in self-management |  |
| 3. EQ5D: Difficulty in activities of daily living |  |
| 4. EQ5D: Pain |  |
| 5. EQ5D: Depressive |  |
| **Activity level** |  |
| 1. Low physical activity (lowest 20%) | 0 = Not low; 1 = Low |
| **Hospitalization history** |  |
| 1. History of hospitalization within 1 year | 0 = No; 1 = Yes |
