# Supplementary material for: Frailty and nutritional inadequacy in older Korean adults: A gender-stratified analysis using National Survey Data
Source: PLoS One. 2025 Oct 27;20(10):e0333620. doi: 10.1371/journal.pone.0333620 (PMC12558530; doi:10.1371/journal.pone.0333620)
Supplement: S2 Table — (DOCX) [file pone.0333620.s002.docx]

S2 Table. Proportions of participants with intakes below the estimated average requirement (EAR) according to frailty groups in men.

|  |  |  | Frailty Groups | | | | | | | |  | | |
| --- | --- | --- | --- | --- | --- | --- | --- | --- | --- | --- | --- | --- | --- |
| Nutrients | KDRIs^a^ | | Non-frail (n=2,469) | Pre-frail (n=2,845) | | | Frail (n=941) | | | *P*-value | | | |
| Proportion of participants with intake below EAR | | |  | | |  | | |  | | | |  |
|  | *Age (y)* | *Men* | *% (standard error)* | | | | | | | | | | |
| Vitamins |  |  |  | |  | | |  | | | |  | |
| Vitamin A | 65 - 74 | 510 μg RAE^b^ | 78.1 (1.0) | 81.9 (0.9) | | | 85.1 (1.3) | | | <.0001 | | | |
|  | 75 or more | 500 μg RAE |  |  | | |  | | |  | | | |
| Thiamine | 65 or more | 0.9 mg | 22.5 (1.0) | 29.8 (1.0) | | | 36.9 (1.8) | | | <.0001 | | | |
| Riboflavin | 65 - 74 | 1.2 mg | 46.1 (1.2) | 58.7 (1.1) | | | 66.8 (1.7) | | | <.0001 | | | |
|  | 75 or more | 1.1 mg |  |  | | |  | | |  | | | |
| Niacin | 65 - 74 | 11 mg NE^c^ | 34.2 (1.2) | 42.6 (1.1) | | | 49.5 (1.9) | | | <.0001 | | | |
|  | 75 or more | 10 mg NE |  |  | | |  | | |  | | | |
| Vitamin C | 65 or more | 75 mg | 61.1 (1.2) | 66.9 (1.1) | | | 75.4 (1.5) | | | <.0001 | | | |
|  |  |  |  |  | | |  | | |  | | | |
| Minerals |  |  |  |  | | |  | | |  | | | |
| Calcium | 65 or more | 600 mg | 67.6 (1.1) | 77.2 (1.0) | | | 79.4 (1.5) | | | <.0001 | | | |
| Phosphorus | 65 or more | 580 mg | 7.6 (0.7) | 11.9 (0.7) | | | 16.9 (1.5) | | | <.0001 | | | |
| Iron | 65 or more | 7 mg | 12.3 (0.8) | 19.7 (0.9) | | | 28.0 (1.8) | | | <.0001 | | | |
| ^a^KDRIs = 2020 Dietary Reference Intakes for Koreans. ^b^RAE = retinol activity equivalent.  ^c^NE = niacin equivalent. Proportion of participants with intake below EAR is presented as percentages with their standard errors (%, s.e.), and p-values were calculated using the Rao-Scott Chi-Square Test. | | | | | | | | | | | | | |
