# Supplementary material for: Frailty and nutritional inadequacy in older Korean adults: A gender-stratified analysis using National Survey Data
Source: PLoS One. 2025 Oct 27;20(10):e0333620. doi: 10.1371/journal.pone.0333620 (PMC12558530; doi:10.1371/journal.pone.0333620)
Supplement: S3 Table — (DOCX) [file pone.0333620.s003.docx]

S3 Table. Proportions of participants with intakes below the estimated average requirement (EAR) according to frailty groups in women.

|  | Frailty Groups | | | | | | | | | | | | |  |  |
| --- | --- | --- | --- | --- | --- | --- | --- | --- | --- | --- | --- | --- | --- | --- | --- |
| Nutrients | KDRIs^a^ | | Non-frail (n=1,818) | | | Pre-frail (n=4,010) | | | Frail (n=2,159) | | | *P*-value | |  |  |
| Proportion of participants with intake below EAR | | |  | |  | | |  | | |  | | |  |  |
|  | *Age (y)* | *Women* | | *% (standard error)* | | | | | | | | | | |  |
| Vitamins |  |  | |  | | |  | | |  | | |  | | |
| Vitamin A | 65 or more | 410 μg RAE^b^ | | 75.9 (1.1) | | | 79.2 (0.8) | | | 82.5 (1.0) | | | <.0001 | | |
| Thiamine | 65 - 74 | 0.8 mg | | 29.6 (1.3) | | | 34.7 (0.9) | | | 43.8 (1.3) | | | <.0001 | | |
|  | 75 or more | 0.7 mg | |  | | |  | | |  | | |  | | |
| Riboflavin | 65 - 74 | 0.9 mg | | 43.7 (1.4) | | | 55.2 (1.0) | | | 65.9 (1.2) | | | <.0001 | | |
|  | 75 or more | 0.8 mg | |  | | |  | | |  | | |  | | |
| Niacin | 65 - 74 | 10 mg NE^c^ | | 49.8 (1.4) | | | 58.0 (1.0) | | | 65.0 (1.2) | | | <.0001 | | |
|  | 75 or more | 9 mg NE | |  | | |  | | |  | | |  | | |
| Vitamin C | 65 or more | 75 mg | | 61.8 (1.4) | | | 68.9 (0.9) | | | 73.7 (1.2) | | | <.0001 | | |
|  |  |  | |  | | |  | | |  | | |  | | |
| Minerals |  |  | |  | | |  | | |  | | |  | | |
| Calcium | 65 or more | 600 mg | | 78.2 (1.2) | | | 83.9 (0.8) | | | 87.7 (0.9) | | | <.0001 | | |
| Phosphorus | 65 or more | 580 mg | | 19.4 (1.2) | | | 28.0 (0.9) | | | 36.9 (1.2) | | | <.0001 | | |
| Iron | 65 - 74 | 6 mg | | 17.6 (1.1) | | | 25.7 (0.9) | | | 33.6 (1.2) | | | <.0001 | | |
|  | 75 or more | 5 mg | |  | | |  | | |  | | |  | | |
| ^a^KDRIs = 2020 Dietary Reference Intakes for Koreans. ^b^RAE = retinol activity equivalent.  ^c^NE = niacin equivalent. Proportion of participants with intake below EAR is presented as percentages with their standard errors (%, s.e.), and p-values were calculated using the Rao-Scott Chi-Square Test. | | | | | | | | | | | | | |  |  |
