# Supplementary material for: Frailty and nutritional inadequacy in older Korean adults: A gender-stratified analysis using National Survey Data
Source: PLoS One. 2025 Oct 27;20(10):e0333620. doi: 10.1371/journal.pone.0333620 (PMC12558530; doi:10.1371/journal.pone.0333620)
Supplement: S4 Table — (DOCX) [file pone.0333620.s004.docx]

※ S4 Table. Proportions of participants with intakes below the tolerable upper intake (UL) according to frailty groups.

|  |  |  | Frailty Groups | | | | | | |
| --- | --- | --- | --- | --- | --- | --- | --- | --- | --- |
|  |  | | Men | | |  | Women | | |
| Nutrients | KDRIs^a^ | | Non-frail (n=2,469) | Pre-frail (n=2,845) | Frail (n=941) |  | Non-frail (n=1,818) | Pre-frail (n=4,010) | Frail (n=2,159) |
| Proportions of participants with intake above UL^b^ | | |  | | | | | | |
|  | *Age (y)* |  | *% (standard error)* | | | | | | |
| Vitamins |  |  |  |  |  |  |  |  |  |
| Vitamin A | 65 or more | 3,000 μg RAE^c^ | 0.5 (0.2) | 0.4 (0.1) | 0.1 (0.1) |  | 0.6 (0.2) | 0.2 (0.1) | 0.3 (0.1) |
| Vitamin C | 65 or more | 2,000 mg | 0 | 0 | 0 |  | 0 | 0.0 (0.0) | 0 |
|  |  |  |  |  |  |  |  |  |  |
| Minerals |  |  |  |  |  |  |  |  |  |
| Calcium | 65 or more | 2,000 mg | 0.7 (0.2) | 0.4 (0.1) | 0.4 (0.2) |  | 0.3 (0.1) | 0.2 (0.1) | 0.2 (0.1) |
| Phosphorus | 65 - 74 | 3,500 mg | 0.1 (0.1) | 0.0 (0.0) | 0 |  | 0.1 (0.1) | 0.0 (0.0) | 0 |
|  | 75 or more | 3,000 mg |  |  |  |  |  |  |  |
| Iron | 65 or more | 45 mg | 1.5 (0.3) | 1.4 (0.2) | 0.7 (0.3) |  | 0.6 (0.2) | 0.9 (0.2) | 1.3 (0.3) |
| ^a^KDRIs = 2020 Dietary Reference Intakes for Koreans. ^b^UL = upper intake. ^c^RAE = retinol activity equivalent. Proportion of participants with intake below EAR is presented as percentages with their standard errors (%, s.e.), and p-values were calculated using the Rao-Scott Chi-Square Test. | | | | | | | | | |
